# Glutamic Acid-Assisted Phytomanagement of Chromium Contaminated Soil by Sunflower (*Helianthus annuus* L.): Morphophysiological and Biochemical Alterations

**DOI:** 10.3389/fpls.2020.01297

**Published:** 2020-09-09

**Authors:** Mujahid Farid, Sheharyaar Farid, Muhammad Zubair, Muhammad Awais Ghani, Muhammad Rizwan, Hafiz Khuzama Ishaq, Saad Alkahtani, Mohamed M. Abdel-Daim, Shafaqat Ali

**Affiliations:** ^1^ Department of Environmental Sciences, University of Gujrat, Gujrat, Pakistan; ^2^ Department of Environmental Sciences and Engineering, Government College University, Faisalabad, Pakistan; ^3^ Department of Chemistry, University of Gujrat, Gujrat, Pakistan; ^4^ Department of Agronomy, University of Agriculture Faisalabad, Institute of Horticultural Science, Faisalabad, Pakistan; ^5^ Department of Zoology, College of Science, King Saud University, Riyadh, Saudi Arabia; ^6^ Pharmacology Department, Faculty of Veterinary Medicine, Suez Canal University, Ismailia, Egypt; ^7^ Department of Biological Sciences and Technology, China Medical University, Taichung, Taiwan

**Keywords:** accumulation, chromium, glutamic acid, photosynthetic pigments, phytoextraction, sunflower

## Abstract

Chelator-assisted phytoremediation is an economical, sustainable, and ecologically friendly method of extracting heavy metals and metalloids from the soil. Organic chelators are thought to enhance metal availability and mobility in contaminated media, thereby improving phytoextraction. The aim of the present study was to examine whether exogenous application of glutamic acid (GA) could improve chromium (Cr) phytoextraction by sunflower plants (*Helianthus annuus* L.). Seeds were planted in plastic pots filled with 5 kg of local agricultural soil spiked with increasing concentrations of Cr (1, 2, and 5 mg kg^−1^). Glutamic acid (5 mM) was applied to soil in solution according to a completely randomized experimental design, and the sunflower plants were harvested after 8 weeks. The results indicated that increasing Cr-induced stress signiﬁcantly inhibited plant growth, leading to reduced biomass, photosynthetic pigment content, activities of antioxidant enzymes, and leaf area of the sunflower plants. However, exogenous addition of GA significantly reduced the Cr-associated toxic effects while also increasing the accumulation of Cr in the plants. Moreover, increasing concentrations of Cr in the soil increased the generation of reactive oxygen species (ROS) responsible for the altered antioxidant enzyme activities. The results revealed that GA application to the topsoil enhanced the Cr concentration and accumulation in the root, stem, and leaves by up to 254, 225, 355, and 47, 59, 150% respectively. Further the GA addition reduced the Cr-induced toxicity in plants and might be helpful for enhancing Cr phytoextraction by sunflower plants.

**Graphical Abstract:**
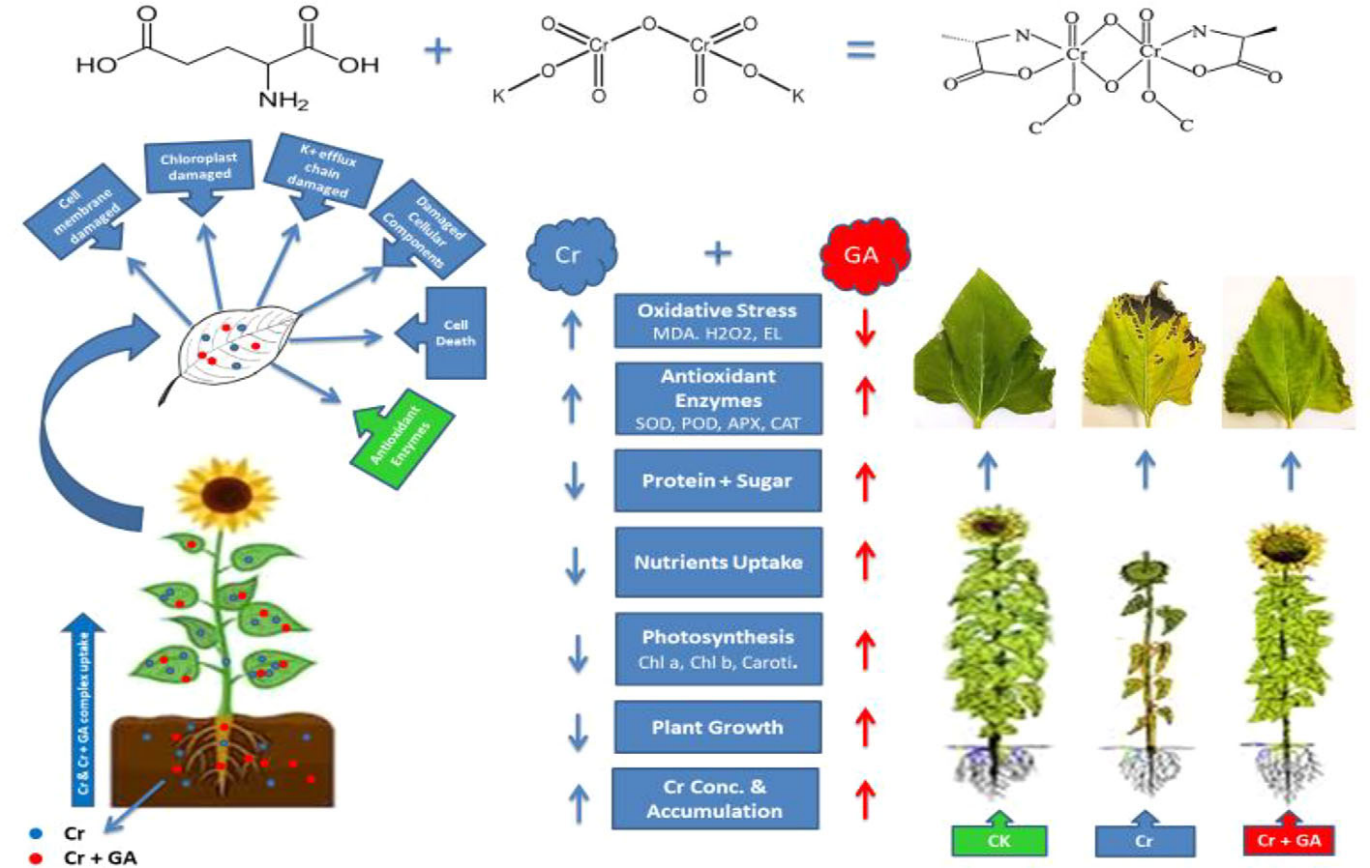


## Introduction

Topsoil provides macro- and micronutrients that are essential for plants and serves as a sink for toxic heavy metals. Among the heavy metals, cadmium (Cd), lead (Pb), chromium (Cr), arsenic (As), and mercury (Hg) are known to be some of the most harmful poisonous elements in the environment (Ali et al., 2013; Adrees et al., 2015a; Shakoor et al., 2014; Ali et al., 2015). Numerous natural and anthropogenic activities result in the escape of hazardous substances into the air, water, and soil that can harm both human health and the environment. Under natural conditions, many crops are vulnerable to a variety of stresses, including those associated with heavy metals, drought, salinity, and disease (Shahid et al., 2017). Heavy metal toxicity is the most common problem affecting plant growth and yield. Heavy metals can also enter the food chain and accumulate in plant and human body tissues (Rizwan et al., 2016). Chromium is a relatively abundant heavy metal in the topsoil owing to its utility in various industrial processes (Farid et al., 2018a). It is used both in the electroplating industry as an anticorrosive and antibiofouling agent, as well as in steel and automobile manufacturing (Farid et al., 2017a). Chromium-mediated toxicity to plants depends on its valence state, with Cr(VI) being very toxic to plants, and Cr(III) less so (Ali et al., 2013; Ali et al., 2015). There is evidence that plants grown on Cr-polluted soil undergo changes in the germination process and exhibit retarded growth (Farid et al., 2017a; Habiba et al., 2018). Additionally, Cr causes many physiological disorders in plants by altering nutrient uptake and the activity of the photosynthetic machinery (Nguyen et al., 2017).

The process of microbial conversion of hexavalent Cr to its trivalent form is cost-effective. The continuous monitoring of microbial activity is essential for this process due to higher sensitivity (Narayania and Shetty, 2012). Although alkaline digestion is used for extracting assimilated and soluble Cr(VI) from soil media (Forte and Mutiti, 2017), this method does not allow for the complete recovery of metals owing to limitations such as the oxidation of Cr(III) (Shahid et al., 2017).

Plants are increasingly used for the remediation of contaminated soil and water as a substitute for the more expensive conventional purifying techniques (Nguyen et al., 2017). Phytoremediation refers to the use of plants for minimizing the concentration of toxic metals in contaminated soil. Plants that are used for phytoremediation and that have an enhanced capacity to accumulate metals from contaminated soil with greater ability to translocate metals in the above ground biomass generally considered as translocation factor greater than 1 (TF > 1) are known as hyperaccumulator plants (Cunningham et al., 1995; Mantry and Patra, 2017). Generally, metals cannot be easily extracted from soil media in great quantities because they form complex bonds with ligands in the soil. Recent studies have shown that plants used for remediation accumulate larger amounts of heavy metals in their roots and then transfer them to above-ground biomass (Wei et al., 2011; Saleem et al., 2015; Lukina et al., 2016). This suggests that plants with greater biomass, such as mustard, rapeseed, maize and sunflower plants, can be beneficial in the field and can survive in the presence of toxic heavy metals (Cekic et al., 2017; Rizwan et al., 2017a). Along with higher biomass plants, many vegetables, ornamental and floating plant species also showed greater accumulation potential (Amir et al., 2020; Khair et al., 2020; Khalid et al., 2020) Phytoremediation refers to the use of plants for the extractive removal of metal contamination from soil and aquatic media. (Salt et al., 1998; USEPA, 2000). An increasing number of studies have shown that specific plant species can remove, degrade, and immobilize a wide range of harmful contaminants (Lasat, 2000). Phytostabilization is used to treat Cr(VI) by converting it into the less toxic Cr(III) with the help of pre-existing plant species (Ali et al., 2013; Farid et al., 2015; Farid et al., 2017b). Different amendments are used worldwide, including organic and inorganic acids (Rizwan et al., 2017a). However, the focus is currently centered on organic acids due to their rapid degradation and potential to enhance metal bioavailability and plant growth (Wiszniewska et al., 2016). Several studies have demonstrated the potential of glutamic acid (GA) as a biodegradable chelating agent (Farid et al., 2018b), such as its ability to enhance the capacity of plants for phytoremediation of different heavy metals (Wei et al., 2011). The success of phytoextraction is mainly reliant on the ability of plants to produce enough aboveground biomass and on their hyperaccumulation potential (translocation of metals from root to shoot) (Yeh and Pan, 2012; Fatima et al., 2020). The results of recent studies have demonstrated the potential of the sunflower plant for the phytoextraction of heavy metals such as Pb, Cd, Cr, Hg, and nickel (Ni) (Yeh and Pan, 2012; De Maria et al., 2013; Farid et al., 2018a; Farid et al., 2018b). Furthermore, the added-value of edible oil yield from the sunflower reduces the costs of phytoremediation (Nehnevajova et al., 2007; 2012). Chemical mutagenesis can also help to increase the capacity of the sunflower to extract heavy metals (Nehnevajova et al., 2009). However, there are only few studies reported on the chelating effect of glutamic acid (GA). Farid et al. (2018a; 2018b) reported that the translocation and accumulation of Cr and Ni were increased in sunflower plants treated with organic chelating acids. Artificially prepared chelators have been extensively used for the plant-mediated extraction of toxic metals in both water and soil media (Huang et al., 1997; Epstein et al., 1999); however, the risks of leaching into groundwater and heavy metal persistence renders them unsuitable for this technique (Farid et al., 2015). The application of gallic acid to the soil of growing sunflower seedlings greatly enhances growth parameters such as oil yield and carbohydrate and protein contents of the plants, as well as the uptake of Ni (Farid et al., 2017b).

The use of GA as a mediator to improve plant growth for enhanced metal extraction has not been comprehensively investigated (Barros-Galvao et al., 2017). Therefore, the aim of the current study was a) to observe the influence of metal concentration on growth characteristics of sunflower (b) to measure the effect of GA on the growth and physiology of sunflower under Cr stress, and (c) to evaluate the phytoextraction potential of sunflower for Cr with AA amendment.

## Materials and Methods

### Plant Resources and Growing Conditions

In the present study, loamy clay soil (52% clay, 26% silt, 22% sand) was collected from the botanical garden of the University of Gujrat, Pakistan, at a depth of 0–20 cm. To eliminate debris and plant residues, the soil was passed through a 2-mm sieve. Healthy seeds of the sunflower (*Helianthus annuus* L.) genotype Faisalabad Hybrid FH-614 were collected from the Oilseeds Research Institute, AARI, Faisalabad (Farid et al., 2017b). Experiments were carried out in the botanical garden of the University of Gujrat under controlled semi-dry weather conditions. The seeds were washed and rinsed with 10% hydrogen peroxide (H_2_O_2_) to remove germs and then rinsed with distilled water. Ten seeds were sown per pot filled with 5 kg of soil, and after 15 days of germination the plants were thinned to five plants per pot to give five experimental replicates. The plants that were removed were then crushed and mixed in the same pot for green manure. The experiment was performed under a complete randomized design (CRD). After 15 and 30 days of germination, each pot was supplied with a 500-ml solution of fertilizer comprising 2.14 g L^−1^ K (as K_2_SO_4_), 2.19 g L^−1^ N [as (NH_2_)_2_CO], and 0.5 g L^−1^ P [as (NH_4_)_2_HPO_4_].

### Treatments

At 4 weeks post-germination, juvenile plants grown in Cr-spiked soil (1, 2, and 5 mg kg^−1^) were treated with GA (5 mM). The following eight combinations consisting five replicated for each treatment were used in the present study: T1, Cr (0 mg kg^−1^) + GA (0 mM); T2, Cr (1 mg kg^−1^); T3, Cr (2 mg kg^−1^); T4, Cr (5 mg kg^−1^); T5, GA (5 mM); T6, Cr (1 mg kg^−1^) + GA (5 mM); T7, Cr (2 mg kg^−1^) + GA (5 mM); T8, Cr (5 mg kg^−1^) + GA (5 mM). The Cr(VI) background level was 0.12 mg kg^−1^ of soil. The GA was dissolved in deionized water and exogenously applied to the soil by pouring into the pot weekly for the following 8 consecutive weeks.

### Plant Sampling and Analysis

After 8 weeks of treatment, three biological replicate plants were harvested to provide a mean for each of the five experimental replicates. All the samples were washed with deionized water, and leftover water was absorbed using a napkin. All the plants were systematically separated into leaves, stems, and roots. Agronomic traits such as root length, plant height, number of flowers and leaves per plant, leaf area, and dry and fresh weight of all parts (leaf, stem, and root) were measured. An electric scale was used for the measurement of fresh and dry biomass. After drying at 90°C in an electric oven, the samples were used for further analysis.

### Plant Accessory Pigment Assay

After 8 weeks of treatment, the fully expanded sunflower leaves were collected to assess the chlorophyll (Chl a, Chl b, total Chl) and carotenoid contents using a spectrophotometer (Halo DB20/DB-20S, Dynamica Labs, London, UK) according to the method of Metzner et al. (1965) with some modifications. Leaf pigments were collected by normalizing with an 85% (v/v) solution of acetone at 4°C with continuous stirring in the dark until staining was completed.

The extracted pigment was centrifuged for 10 min at 4,000 rpm, and the resulting pigment was placed in a spectrophotometer to record the absorbance at the wavelengths of 663, 644, and 452.5 nm against a blank of 85% acetone (v/v) solution. To estimate chlorophyll a and b and carotenoid contents, the results were integrated according to the adjusted extinction constants and the equation given by Lichtenthaler (1987) as follows:

$$\text{Chlorophyll a }({\text{mg kg}}^{-1})=10.3\times E663-0.98b\times bE644$$

$$\text{Chlorophyll b}({\text{mg kg}}^{-1})=19.7\times E644-3.87b\times bE663$$

Total chlorophyll=chlorophyll a+chlorophyll b

$$\text{Total carotenoids }({\text{mg kg}}^{-1})=4.2\times \text{E}452.5-\{(0.0264\times \text{chlorophyll a})+(0.426\times \text{chlorophyll b})\}$$

The pigment quantities were expressed as milligrams per gram fresh weight.

### Analysis of Chromium Content

A known weight of leaves, stems, or roots samples dehydrated at 90°C was placed in a muffle-type furnace for 7 h at 650°C then the remnants were mixed with nitric acid (HNO_3_) and hydrochloric acid (HCl) at a 3:1 ratio, and the samples were then diluted to 50 ml with deionized water and examined using an atomic absorption spectrometer (NOVA A400 Analytik Jena, Germany) to measure the Cr concentration, as described by Ehsan et al. (2013).

The amount of Cr was calculated as follows:

$${\text{Cr concentration (mg kg}}^{-1}{\text{) = metal reading of digested sample (mg L}}^{-1})\times \text{ dilution factor}$$

Cr accumulation was measured with the following formula:

$${\text{Cr accumulation (mg plant}}^{-1}{\text{) = Cr concentration in each organ (mg kg}}^{-1})\times \text{dry weight of the organ (kg)}$$

### Determination of Electrolyte Leakage and Levels of MDA and H_2_O_2_

Electrolyte leakage (EL) was estimated using a technique defined by Dionisio-Sese and Tobita (1998). After 8 weeks of treatment, the uppermost and fully stretched sunflower leaves were cut into 5-mm long pieces and then inserted into test tubes filled with 8 ml of deionized water. The initial electrical conductivity (EC1) was then measured after incubation in a water bath at 32°C for 2 h. To discharge all the electrolytes into the solution, the test tubes containing the same samples as of EC1 were first autoclaved at 121°C for 20 min; when the samples had cooled to room temperature (25°C), the second electrical conductivity (EC2) was measured using a pH/conductivity meter (INCO-LAB, Model 720, Kuwait). The following equation was used to compute EL:

EL = (EC1/EC2)×100

The malondialdehyde (MDA) concentration in the roots and leaves of the sunflower was determined by the thiobarbituric acid (TBA) reaction method (Heath and Packer, 1968) modified by Dhindsa et al. (1981) and Zhang and Kirkham (1994). Root and leaf samples (0.25 g) were homogenized with 5 ml (0.1%) of trichloroacetic acid (TCA). The resulting mixture was centrifuged at 10,000 × *g* for 5 min, and an additional 4 ml of TCA (20%) containing TBA (0.5%) was mixed into 1 ml of the supernatant solution. The solution was heated for 30 min at 95°C and subsequently cooled in an ice bath. The post-centrifugation absorbance at 600 nm was subtracted from the absorbance at 532 nm of the same mixture. An extinction coefficient of 155 mM^−1^ cm^−1^ was used to compute MDA content.

The H_2_O_2_ concentration was measured using the colorimetric method of Jana and Choudhuri, 1981. To extract the H_2_O_2_, leaf and root samples (50 mg) were homogenized with 3 ml of 50 mM phosphate buffer (pH 6.5) and subsequently centrifuged at 6,000 × *g* for 25 min. To measure the concentration of H_2_O_2_, the supernatant was mixed with 1 ml of titanium sulfate (0.1%) and 20% H_2_SO_4_ (v/v), and 3 ml of the solution was again centrifuged at 6000 × *g* for 15 min until the supernatant turned yellow. The supernatant was subsequently analyzed at 410 nm to determine the concentration of H_2_O_2_ which was calculated using the extinction coefficient 0.28 µmol^−1^ cm^−1^.

### SPAD Value

One week before plant harvest, leaf greenness (chlorophyll) was measured in the second uppermost fully expanded leaf using a SPAD chlorophyll meter SPAD-502 (Zhejiang Top Cloud-Agri Technology Co., Ltd.).

### Evaluation of Antioxidant Enzymes and Protein Content

The contents of antioxidant enzymes (SOD, POD, APX, and CAT) in the roots and leaves were determined using a UV-Visible spectrophotometer (Halo DB20/DB-20S, Dynamica Labs, London, UK). Samples of roots and fully expanded leaves were collected after 8 weeks of treatment to measure the enzyme content. Leaf and root samples (1 g) were snap-frozen in liquid nitrogen and ground using a precooled mortar and pestle and then mixed with 0.05 M phosphate buffer (pH 7.8). The prepared samples were then filtered through four layers of muslin cloth and the homogenates centrifuged at 12,000 × *g* for 10 min at 4°C. The final supernatant was directly used to measure the SOD and POD contents according to the method described by Zhang (1992). The same supernatant was used for measurement of the soluble protein content using standard albumin and dye (Coomassie brilliant blue G-250) as reported by Bradford (Bradford, 1976).

The CAT (EC 1.11.1.6) content was measured following the protocol of Aebi (1984). The assay mixture was prepared by adding 100 μl of the extract of each enzyme and H_2_O_2_ (300 mM) to 2.8 ml of 50 mM phosphate buffer (2 mM citric acid (CA), pH 7.0). The CAT concentration was recorded by measuring the decrease in absorbance at 240 nm as a consequence of the consumption of H_2_O_2_ (*ϵ* = 39.4 mM^−1^ cm^−1^). Similarly, APX (EC 1.11.1.11) content was assessed by mixing 100 μl of enzyme extract, 100 μl of H_2_O_2_ (300 mM), 2.7 ml of 25 mM potassium phosphate buffer (2 mM CA, pH 7.0), and 100 μl of ascorbic acid (7.5 mM) (Nakano and Asada, 1981). The oxidation pattern of ascorbate was estimated from the variations in wavelength at 290 nm (*ϵ* = 2.8 mM^−1^ cm^−1^).

### Statistical Analysis

Data are expressed as the average values of five replicates for each treatment. One-way Analysis-of-variance (ANOVA) followed by Tukey’s *post-hoc* test, and significant differences were calculated by all pairwise comparison to determine significant differences and standard deviation (SD). The different small letters in figures and tables describe values that are significantly different at p ≤ 0.05. All analyses were carried out using Statistix 10.0 software.

## Results

### Plant Agronomic Traits

Significant variation was observed in biomass and growth parameters, including fresh and dry mass of the root, stem, and leaf, along with root length, plant height, leaf area, leaf number, and total number of flowers (**Table 1**). All the plants showed reduced growth and impaired agronomic traits resulting from Cr toxicity. Compared with controls, the most severe effects were seen at the highest concentration of Cr used (5 mg kg^−1^).

**Table 1:** Effect of different concentrations of Cr alone and/or in combination with GA on agronomic traits of sunflower.

Treatments	Cr Concentration (mg kg^−1^)				Cr Concentration (mg kg^−1^)				
	Cr 0	Cr 1	Cr 2	Cr 5	Cr 0	Cr 1		Cr 2	Cr 5
	**Root Fresh Weight (g)**				**Stem Fresh Weight (g)**				
GA 0	21.43 ± 1.17a	14.50 ± 1.32c	10.83 ± 0.76d	6.16 ± 1.52e	50.59 ± 1.52a	31.96 ± 0.51bc	24.13 ± 2.80de		12.4 ± 0.86f
GA 5 mM	22.36 ± 1.20a	17.60 ± 0.65b	14.43 ± 0.51c	10.93 ± 0.95d	51.73 ± 2.41a	39.13 ± 0.80b	30.00 ± 1.30cd		20.60 ± 1.60ef
	**Root Dry Weight (g)**				**Stem Dry Weight (g)**				
GA 0	7.90 ± 0.52a	5.06 ± 0.20c	2.76 ± 0.51d	2.10 ± 0.1e	17.56 ± 1.00a	11.26 ± 0.60c	9.06 ± 0.40de		5.49 ± 0.50f
GA 5 mM	8.26 ± 0.25a	6.36 ± 0.40b	5.13 ± 0.15c	3.93 ± 0.30d	16.36 ± 0.70a	14.10 ± 0.55b	10.73 ± 0.40cd		8.43 ± 0.40e
	**Leaf Fresh Weight (g)**				**Leaf Dry Weight (g)**				
GA 0	24.76 ± 1.50a	17.20 ± 1.25bc	12.00 ± 1.94d	7.60 ± 1.49e	7.86 ± 0.15a	5.26 ± 0.25c	4.23 ± 0.25d		3.03 ± 0.05e
GA 5 mM	24.43 ± 0.75a	20.63 ± 0.70b	16.00 ± 0.50c	11.00 ± 1.50de	7.9 ± 0.10a	6.86 ± 0.15b	4.56 ± 0.40cd		4.66 ± 0.49cd
	**Plant Height (cm)**				**Root Length (cm)**				
GA 0	99.16 ± 5.20a	71.6 ± 3.04c	55.50 ± 2.68d	36.63 ± 5.29e	35.73 ± 2.27a	24.76 ± 3.95bc	19.7 ± 1.44cd		10.13 ± 1.04e
GA 5 mM	101.5 ± 3.77a	86.21 ± 3.43b	78.13 ± 6.19bc	56.9 ± 2.50d	36.46 ± 0.60a	30.5 ± 1.93ab	25.03 ± 3.06bc		14.23 ± 1.44de
	**No. of Leaves Plant^−1^**				**No. of Flowers Plant^−1^**				
GA 0	16.66 ± 1.15b	13.66 ± 0.57c	8.33 ± 0.57e	7.00 ± 0.00e	8.66 ± 0.57a	6.16 ± 0.57bc	4.34 ± 0.57d		2.00 ± 0.00e
GA 5 mM	19.00 ± 1.00a	16.00 ± 1.00b	13.33 ± 0.57cd	11.33 ± 0.57d	8.00 ± 1.00a	7.34 ± 0.57ab	5.34 ± 0.57cd		3.67 ± 0.57de
	**Leaf Area (cm^2^)**								
GA 0	154.37 ± 6.24a	124.03 ± 13.4b	96.56 ± 2.21c	75.4 ± 5.04d					
GA 5 mM	156.13 ± 1.97a	140.1 ± 4.41ab	125.90 ± 2.40b	88.05 ± 5.28cd					

Values are means of five replicates ± S.D. Mean values followed by small different letters are significantly different from each-others at P ≤ 0.05.

Chromium application at 5 mg kg^−1^ reduced root length, plant height, and leaf area by 71, 63, and 51%, respectively, when compared with controls. Similarly, fresh and dry mass of the leaf, stem, and root declined by 69 and 61%, 73 and 68%, and 68 and 73%, respectively, compared with control. However, GA application reduced the Cr-induced toxicity and exerted an ameliorative effect on the morphology of the sunflower plants (**Table 1**). The addition of GA (5 mM) alone or with Cr (1, 2, or 5 mg kg^−1^) significantly improved the agronomic traits of the plants. Root length (40%) and plant height (55%) were both increased under combined GA (5 mM) and Cr (5 mg kg^−1^) treatment when compared with Cr treatment alone (5 mg kg^−1^). A similar trend was observed for fresh and dry biomass of the leaf, stem, and root (44 and 54%, 53 and 53%, and 77 and 87%, respectively) under T8 (5 mg kg^−1^ Cr and 5 mM GA) when compared with the respective controls.

### Plant Accessory Pigments

**Figure 1:**
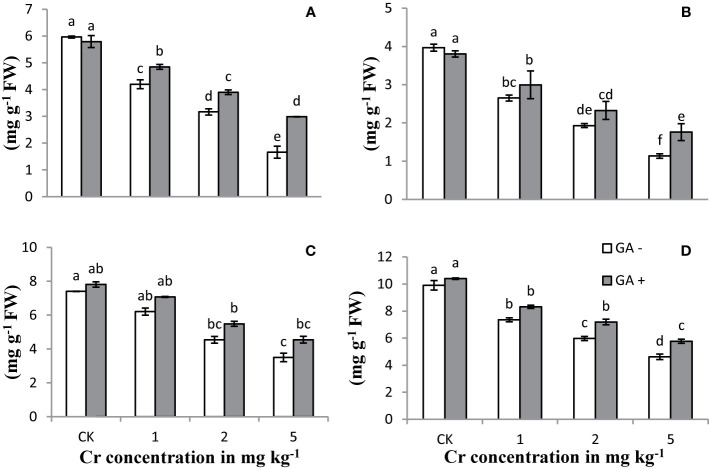
Effect of Cr and GA on chlorophyll a **(A)**, chlorophyll b **(B)**, total chlorophylls **(C)** and carotenoids **(D)** in Sunflower grown in soil with increasing Cr concentrations (0, 1, 2 and 5 mg kg^−1^) and GA (0 and 5mM). Values are demonstrated as means of five replicates along with standard deviation. Different small letters indicate that values are signiﬁcantly different at *P < 0.05*.

Increasing the Cr concentration in the treatments led to a significant reduction in the chlorophyll and carotenoid contents compared with the controls (**Figure 1**). Extreme reductions of 82 and 71% in the carotenoid and total chlorophyll contents, respectively, were observed with Cr addition at 5 mg kg^−1^ when compared with the controls. Co-amendment of GA and Cr decreased the Cr-induced toxicity in terms of improved chlorophyll and carotenoid contents. Maximum increases were observed in Chl a (79%), Chl b (55%), total Chl (69%), and carotenoid (109%) contents under T8 (5 mg kg^−1^ Cr and 5 mM GA). Plants co-treated with Cr and GA showed greater potential to tolerate Cr stress, as evidenced by their ability to retain greater amounts of carotenoid and chlorophyll contents.

### Soluble Protein and SPAD Value

**Figure 2:**
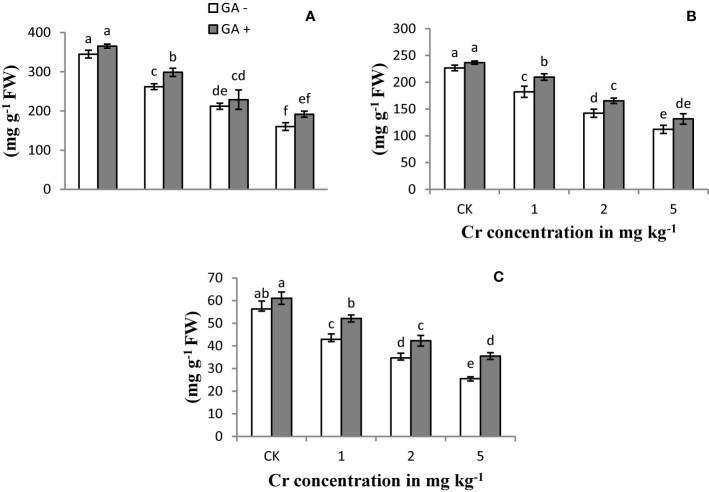
Effect of Cr and GA on SP in leaves **(A)**, SP in root **(B)** and SPAD value **(C)** in Sunflower grown in soil with increasing Cr concentrations (0, 1, 2 and 5 mg kg^−1^) and GA (0 and 5 mM). Values are demonstrated as means of five replicates along with standard deviation. Different small letters indicate that values are signiﬁcantly different at *P < 0.05*.

Soluble protein content in the leaf and root and the SPAD value of the sunflower plants were significantly reduced with increasing Cr concentrations compared with the control (**Figure 2**). At the higher level of Cr used (5 mg kg^−1^), the protein content in the root and leaf was reduced by 74 and 38%, respectively, compared with the control samples. In addition, the greatest decrease in the SPAD value (68%) was recorded at the highest Cr concentration. The application of GA resulted in a gradual improvement in the SPAD rate and the protein content in the leaves and roots under Cr-induced stress conditions. Glutamic acid treatment led to increases in the soluble protein content of roots and leaves of 40 and 20%, receptively, under 5 mg kg^−1^ Cr treatment. Similarly, the maximum SPAD value also increased by 60% with T8 (5 mg kg^−1^ Cr and 5 mM GA) compared with the other GA treatments.

### Electrolyte Leakage and MDA and Hydrogen Peroxide Content

**Figure 3:**
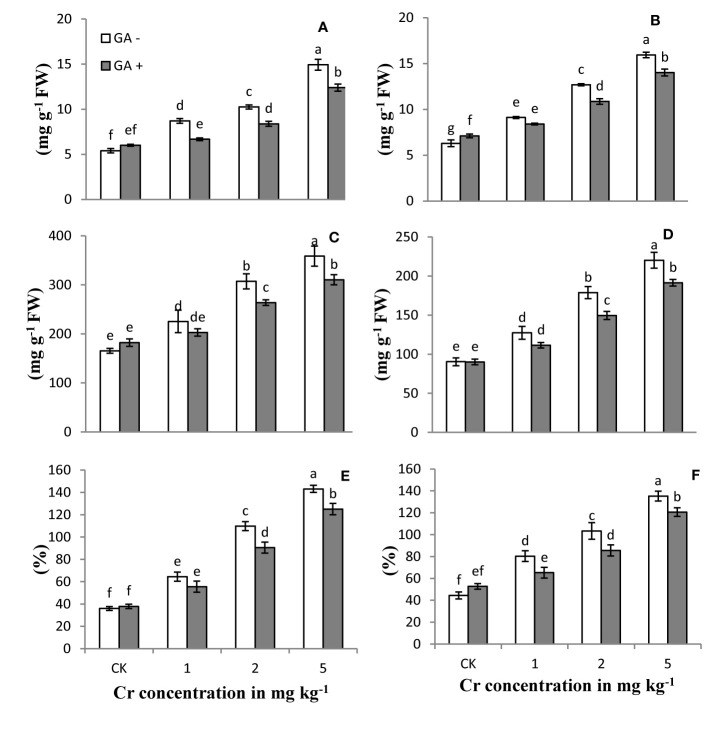
Effect of Cr and GA on MDA in root **(A),** MDA in leaves **(B),** H_2_O_2_ in root **(C),** H_2_O_2_ in leaves **(D),** EL in roots **(E)** and EL in leaves **(F)** of Sunflower grown in soil with increasing Cr concentrations (0, 1, 2 and 5 mg kg^-1^) and GA (0 and 5mM). Values are demonstrated as means of five replicates along with standard deviation. Different small letters indicate that values are signiﬁcantly different at *P<0.05*.

The levels of reactive oxygen species (ROS) and EL in the roots and leaves of the sunflower plants treated with Cr and/or GA are shown in **Figure 3**. H_2_O_2_ and MDA content and EL of the plants increased with increasing Cr concentrations (1, 2, and 5 mg kg^−1^) compared with control. The largest increases in H_2_O_2_, MDA content, and EL were 143, 153, and 148% in leaves and 108, 176, and 116% in roots, respectively, in soils treated with Cr (5 mg kg^−1^). Glutamic acid application significantly attenuated the Cr-induced oxidative stress in both roots and leaves by reducing ROS production at all Cr levels tested, while a slight increase was also noted in control plants under GA treatment (**Figure 3**). Under combined Cr and GA treatment, the major decrease in H_2_O_2_ generation was 16% in leaves and 14% in roots in soil treated with 2 mg kg^−1^ Cr; for MDA production, the greatest decrease was 14% in leaves with 2 mg kg^−1^ Cr treatment and 23% in roots with 1 mg kg^−1^ at Cr; and for EL, the maximum reduction was 17% in leaves with 2 mg kg^−1^ Cr and 18% in roots at 1 mg kg^−1^ Cr.

### Antioxidant Enzyme Activities

**Figure 4:**
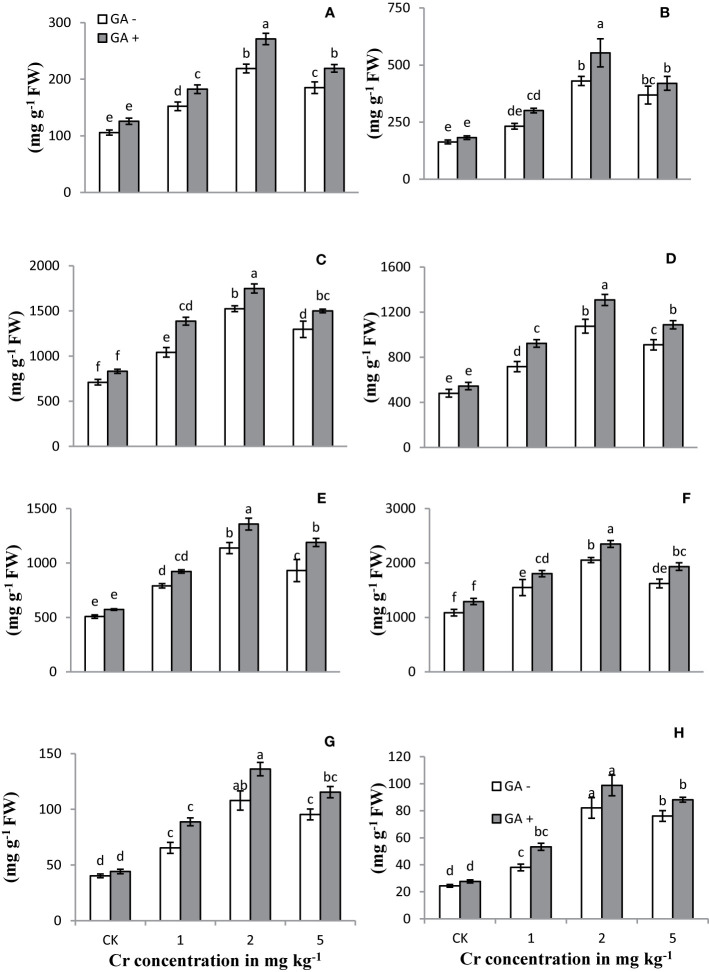
Effect of Cr and GA on APX in root **(A)**, APX in leaves **(B)**, CAT in root **(C)**, CAT in leaves **(D)**, POD in root **(E)**, POD in leaves **(F)**, SOD in root **(G)** and SOD in leaves **(H)** of Sunflower grown in soil with increasing Cr concentrations (0, 1, 2 and 5 mg kg^−1^) and GA (0 and 5 mM). Values are demonstrated as means of five replicates along with standard deviation. Different small letters indicate that values are signiﬁcantly different at *P < 0.05*.

The activities/concentrations of antioxidant enzymes, including APX, CAT, POD, and SOD, in both leaves and roots were measured under Cr and GA co-application (**Figure 4**). Compared with the control, soil spiked with 1 and 2 mg kg^−1^ Cr significantly improved the activities of all antioxidant enzymes in both roots and leaves (231 and 311% for SOD; 124 and 88% for POD; 106 and 163% for APX; and 114 and 123% for CAT, respectively). At the highest concentration of Cr tested (5 mg kg^−1^), the activities of these enzymes tended to decrease in both roots and leaves (26 and 34% for SOD; 18 and 20% for POD; 15 and 14% for APX; and 14 and 15% for CAT, respectively) compared with Cr treatment at 2 mg kg^−1^. Glutamic acid application further increased the activities of these antioxidant enzymes and exerted an additive effect under Cr stress. The greatest antioxidant enzyme activities were observed in both roots and leaves of the sunflower when GA was co-applied with 2 mg kg^−1^ Cr. Under combined Cr and GA treatment, the largest increase in SOD activity was 15% in roots and 31% in leaves at 1 mg kg^−1^ Cr; for POD activity, the largest increase was 27% in roots and 19% in leaves at 5 mg kg^−1^ Cr; for APX activity, the largest increase was 23% in roots at 2 mg kg^−1^ Cr and 29% in leaves at 1 mg kg^−1^ Cr; and for CAT activity, the largest increase was 33% in roots and 28% in leaves at 1 mg kg^−1^ Cr.

### Chromium Uptake

Chromium addition at the concentrations of 1, 2, and 5 mg kg^−1^ significantly enhanced the Cr concentration and accumulation in the roots, stems, and leaves of the sunflower plants (**Table 2**). Trace levels of Cr were detected in control plants, likely due to background Cr concentrations in the soil. The highest concentration and accumulation of Cr in the root, stem, and leaf were observed at the greatest concentration of Cr tested (5 mg kg^−1^) (increases of 254, 225, 355 and 47, 59, 150%, respectively, compared with Cr treatment at 1 mg kg^−1^). The combined GA and Cr treatment further markedly enhanced the absorption and accumulation of Cr in the root, stem, and leaf of the sunflower plant. At the highest concentrations of Cr (5 mg kg^−1^) and GA (5mM), the concentration and accumulation increased by 24 and 132% in the root, 31 and 59% in the stem, and 19 and 150% in the leaf, respectively.

**Table 2:** Effect of different concentrations of Cr alone and/or in combination with GA on Cr concentration and accumulation in sunflower.

Treatments	Cr Concentration (mg kg^−1^)				Cr accumulation (µg plant^−1^)	
	Leaf	Stem	Root	Leaf	Stem	Root
CK	2.06 ± 0.15g	10.71 ± 3.12f	13.53 ± 0.51g	18.40 ± 1.32f	190.41 ± 67.44e	106.88 ± 6.90e
GA	3.16 ± 0.04g	12.66 ± 2.08f	22.48 ± 0.54g	29.18 ± 0.225f	207.90 ± 40.88e	185.82 ± 3.79e
Cr 1	62.46 ± 8.19f	142.15 ± 13.01e	175.49 ± 12.78f	328.99 ± 50.77e	1598.03 ± 101.17d	887.70 ± 39.70d
Cr 1+ GA	100.50 ± 5.25e	189.36 ± 13.24e	237.33 ± 24.96e	690.10 ± 28.02cd	2667.36 ± 196.17c	1506.29 ± 116.88c
Cr 2	151.24 ± 3.11d	258.33 ± 17.23d	360.01 ± 17.32d	640.24 ± 32.23d	2344.83 ± 237.35c	1360.33 ± 238.03c
Cr 2+GA	193.83 ± 12.98c	378.33 ± 17.55c	478.33 ± 17.55c	885.17 ± 54.92b	4071.50 ± 533.58b	2457.16 ± 161.94b
Cr 5	271.76 ± 10.52b	462.33 ± 42.85b	622.33 ± 30.27b	824.35 ± 28.94bc	2545.16 ± 359.40c	1307.66 ± 106.20cd
Cr 5+GA	325.61 ± 15.01a	606.10 ± 24.24a	772.66 ± 24.07a	1519.54 ± 105.67a	5104.33 ± 80.50a	3043.33 ± 328.51a

Values are means of five replicates ± S.D. Mean values followed by small different letters are significantly different from each-others at P ≤ 0.05.

## Discussion

### Agronomic Traits

The increased Cr concentrations led to a significant decrease in the growth of the sunflower plants (**Table 1**). Several studies regarding Cr-induced toxic effects on sunflower plant have been widely reported (Farid et al., 2017a; Farid et al., 2018a; Farid et al., 2018b). Chromium-induced toxicity was shown to severely impair nutrient uptake in plants, leading to reduced biomass production and stunted growth (Tauqeer et al., 2016; Fozia et al., 2008). Similarly, Cr stress has been reported to inhibit the growth (Atta et al., 2013) and reduce the fresh and dry biomass of sunflower plants (Saleem et al., 2015). In addition to Cr, sunflower plants also show suppressed growth under Ni (Lin et al., 2003), Ag (Farid et al., 2018a), Cd (Junior et al., 2015), As (Farid et al., 2016), and Cu stress (Imran et al., 2013). Chromium-associated toxicity has also been reported in different plants such as *Braccisa napus* L. (Afshan et al., 2015), *Pisum sativum* L. (Tripathi et al., 2015), *Lemna minor* L. (Sallah-Ud-Din et al., 2017), *Echinochloa colona* (Rout et al., 2000), pea (Rodriguez et al., 2012), and wheat (Ali B. et al., 2015; Mathur et al., 2016). A significant reduction in the quality and yield has been observed in carrots grown on Cr-contaminated soil exceeding the permissible limit (Ding et al., 2014). In our study, plants treated with GA showed improved growth and greater biomass when compared with those treated with Cr alone. Moreover, at the greatest Cr concentrations tested, GA application alleviated the Cr-induced stress in the sunflower. The growth- moderating role of GA under cold stress for rice (Jia et al., 2017) and under drought stress for wheat (Liu et al., 2011) has previously been reported. The addition of GA was shown to regulate plant nutrient uptake and translocation by enhancing nutrient availability (Farid et al., 2018b). As depicted in **Table 1**, GA exerted a growth-moderating effect on sunflower plants at all Cr concentrations applied. Similar stress-alleviating effects were observed when GA was applied to sunflower plants under Ag stress (Farid et al., 2018a). This growth-moderating role of GA is very similar to that of other organic chelators such as citric acid, aminolaevulinic acid, and abscisic acid (Barros-Galvao et al., 2017; Farid et al., 2018a; Farid et al., 2018b). In the present study, we confirmed the growth- moderating and growth-regulatory effects of GA on sunflower plants under heavy metal stress.

### Chlorophyll Content

In plants, Cr-related toxicity is similar to that of Ag, Ni, Cd, Pb, as well as other metals (Habiba et al., 2015; Farooq et al., 2016; Farid et al., 2018a). The increasing concentrations of Cr applied and the associated toxicity significantly reduced the performance of the photosynthetic machinery (**Figure 1**). This decrease in chlorophyll and carotenoid contents in sunflower plants is in agreement with the results reported by Farid et al. (2017a) and Fozia et al. (2008). Atta et al. (2013) and Saleem et al. (2015) both reported that Cr stress can disrupt water translocation and cause nutrient deficiency in plants, whereas Singh et al. (2013) and Najeeb et al. (2011) described that reduced chlorophyll and carotenoid content might result from an impaired electron chain due to the deterioration of the photosynthetic system. Disruption of the photosynthetic machinery linked to Cr toxicity has also been reported in wheat (Jabeen et al., 2016), *Brassica napus* (Afshan et al., 2015), *Lemna minor* (Sallah-Ud-Din et al., 2017), and mung bean (Adrees et al., 2015a). In the current study, the decreased levels of carotenoids and photosynthetic pigments might be a consequence of exacerbated electrolyte leakage and ROS generation (**Figure 3**). The results clearly demonstrated that the introduction of GA markedly improved the Cr-associated toxic effects and the functioning of the photosynthetic machinery of the plants, an idea that is supported by the similar findings of Farid et al. (2018a). Forde et al. (2007) also reported that the chlorophyll and carotenoid contents were increased in plants under GA supply.

### Soluble Protein and SPAD

Both the soluble protein content and SPAD value of the sunflower decreased in the leaves and roots with increasing Cr concentrations (**Figure 2**). Our findings are in line with the conclusions of Farid et al. (2017a; 2018a; 2018b) and Saleem et al. (2015) who reported a decline in soluble protein content and SPAD value of sunflower plants exposed to Cr stress. In agreement with the present findings, many plant species also show reduced soluble protein content and SPAD values, such as cotton exposed to Pb (Anwaar et al., 2015), wheat exposed to Cr (Adrees et al., 2015b), and maize and mung bean exposed to Cd (Hussain et al., 2006; Sabir et al., 2014). Rizwan et al. (2017a) stated that the SPAD value is directly associated with the leaf chlorophyll content, suggesting that a decrease in chlorophyll content and water translocation might be responsible for the observed decrease in the SPAD value. The results indicated that the application of GA alone or in combination with Cr greatly increased the soluble protein content and SPAD value (**Figure 3**), which was in close agreement with the results of studies by Jia et al. (2017) and Farid et al. (2018a). Increased enzymatic activities (**Figure 4**) are the indication of damage to the soluble protein levels in plant (Forde and Mutiti, 2017). Increased soluble protein content under GA application has been observed for plants under different heavy metal stresses, such as Cd, Cu, Pb, and Zn, as reported by Doumett et al. (2010).

### Reactive Oxygen Species

Exposure of sunflower plants to Cr resulted in oxidative damage, which was attributable to electrolyte leakage and ROS generation (**Figure 4**; Farid et al., 2018a; Farid et al., 2018b). Ahmad et al. (2017) also reported oxidative damage in sunflower plants exposed to Cr stress. This response of sunflower plants to stress induced by various heavy metals is concisely reviewed by Rizwan et al. (2017b). Similar to sunflower plants, *Brassica napus*, soybean, barley, and wheat also show higher ROS production under Cr stress (Adrees et al., 2015b; Gill et al., 2016). Chromium-induced toxicity was shown to alter K^+^ efflux and impair the electron transport chain in plants, leading to increased production of OH^−^ and O^−^ free radicals, further enhancing EL (Fozia et al., 2008; Rizwan et al., 2017a). Recent studies reported that high levels of EL and ROS generation were observed in plants exposed to biotic and abiotic stresses, such as salinity (Noman et al., 2015), drought (Arshad et al., 2016), pathogen attack (Giovanini et al., 2006), and herbicide application (Song et al., 2007). Gallic acid application to soil reduced EL and ROS generation in plants under Cr stress. This decrease might be due to the activation of the antioxidant defense system and repair of the plant electron transport chain and water translocation system, similar to the findings of Sallah-Ud-Din et al. (2017). Furthermore, GA is reported to act like other organic chelators in scavenging ROS by improving the natural protection system of plants. In this study, the improvements in plant growth, biomass, and photosynthetic machinery might have resulted from decreased EL and ROS production caused by the addition of GA.

### Antioxidant Defense System

The sunflower is known to activate its antioxidant enzyme defense system under biotic and abiotic stresses (Farid et al., 2018a). Activation of these enzymes was also observed in the current study (**Figure 4**). Increasing the Cr concentration significantly enhanced the activities of POD, SOD, CAT, and APX. Interestingly, at the highest Cr concentration used (5 mg kg^−1^), the activities of these enzymes tended to decrease. This behavior of the sunflower plant under Cr stress has also been previously reported by Farid et al. (2017a; 2018a; 2018b). Additionally, the sunflower also showed a similar trend under Pb, Cu, and Cd stress (Li et al., 2011; Shamsi et al., 2014). Rizwan et al. (2017a; 2017b) reported an increase in antioxidant enzyme activity resulting from ROS production under heavy metal stress. Demidchik (2012) reported that plants activate the ROS scavenging defense system by regulating the electron transport chain and K^+^ efflux. At lower Cr concentrations, antioxidant enzymes favored normal plant activity; however, the antioxidant defense system could not cope with the higher Cr concentrations. This phenomenon has been observed in sunflower plants under the stress of various heavy metals, as summarized by Rizwan et al. (2017a), as well as in sunflower plants under drought, salinity, and heat stress (Naeem et al., 2012; Kanto et al., 2015). Similar to that observed with other organic acids, GA application led to a significant increase in the activities of all antioxidant enzymes in sunflower plants supplemented with Cr (Sallah-Ud-Din et al., 2017; Farid et al., 2018a).

### Chromium Concentration and Accumulation

The sunflower plant is widely reported to be a hyperaccumulator plant for various heavy metals due to its height and root structure (Farid et al., 2017a, Rizwan et al., 2017a; Rizwan et al., 2017b; Farid et al., 2018a; Farid et al., 2018b). In this study, Cr accumulation gradually increased in all the plant organs with increasing Cr content in the soil (**Table 2**), which is similar to the findings reported by Fozia et al. (2008) and Saleem et al. (2015). Several studies have reported that Cr can be readily absorbed by different plants, including *Lemna minor* (Sallah-Ud-Din et al., 2017), *Brassica napus* (Afshan et al., 2015), wheat (Adrees et al., 2015b), and mung bean (Jabeen et al., 2016). Chromium uptake and accumulation are mainly dependent on its availability and mobility in the soil (Ali et al., 2011; Ali et al., 2013) and can be affected by the addition of exogenously applied chelators (Shahid et al., 2017). Similarly, the addition of GA under Cr stress significantly enhanced Cr uptake and accumulation in our study. This increased accumulation could be attributed to the chelating properties of GA, which has the ability to detach metals from organic fractions in the soil as reported by Doumett et al. (2010). The chelating properties of GA have also been reported by Farid et al. (2018a) and Jia et al. (2017). The greater the plant biomass, the higher the accumulation of metal in plant tissues. Relatively few studies have investigated the chelating ability of GA for heavy metals and the response of the sunflower at the genetic and molecular levels. Our results were broadly in agreement with our stated hypothesis that GA can enhance metal accumulation, growth, and biomass in plants.

### Conclusions

The results of the present study indicated that sunflower growth, biomass, and biochemical attributes were significantly reduced as a result of Cr-induced toxicity. Increasing the concentration of applied Cr significantly increased Cr concentration and accumulation in the root, stem and leaf of sunflower plant. Meanwhile, we also found a positive role of GA due to its ability to regulate normal functioning and plant growth. Glutamic acid markedly ameliorated the Cr-induced toxicity in the sunflower, improving its morphological, physiological, and biochemical attributes. Our results indicated that the sunflower might be a suitable and potential candidate for Cr phytoextraction under GA application in Cr contaminated soils. Future studies are required to elucidate the associated molecular and genetic mechanisms.

## Data Availability Statement

The raw data supporting the conclusions of this article will be made available by the authors, without undue reservation, to any qualified researcher.

## Author Contributions

All authors contributed to the article and approved the submitted version.

## Funding

This work was funded by Researchers Supporting Project number (RSP 2020/26), King Saud University, Riyadh, Saudi Arabia.

## Conflict of Interest

The authors declare that the research was conducted in the absence of any commercial or financial relationships that could be construed as a potential conflict of interest.
